# Combination of targeted pharmacotherapy and immunotherapy with anti‐CD19 CAR NK cells in acute lymphoblastic leukemia

**DOI:** 10.1002/hem3.70238

**Published:** 2025-10-15

**Authors:** Hanna Kirchhoff, Caroline Schoenherr, Lisa Fleischer, Elizabeth K. Schweighart, Ruth Esser, Steven R. Talbot, Axel Schambach, Ulrike Koehl, Olaf Heidenreich, Matthias Eder, Michaela Scherr

**Affiliations:** ^1^ Department of Hematology, Hemostasis, Oncology and Stem Cell Transplantation Hannover Medical School Hannover Germany; ^2^ Princess Maxima Center for Pediatric Oncology Utrecht The Netherlands; ^3^ Hannover Medical School Institute of Cellular Therapeutics (ICT) Hannover Germany; ^4^ Hannover Medical School Institute for Laboratory Animal Science Hannover Germany; ^5^ Hannover Medical School Institute of Experimental Hematology Hannover Germany; ^6^ Hannover Medical School REBIRTH‐Research Center for Translational Regenerative Medicine Hannover Germany; ^7^ Division of Hematology/Oncology, Boston Children's Hospital Harvard Medical School Boston Massachusetts USA; ^8^ Fraunhofer Institute for Cell Therapy and Immunology (IZI) Leipzig Germany; ^9^ Institute of Clinical Immunology University of Leipzig Leipzig Germany; ^10^ Department of Hematology University Medical Centrum Utrecht Utrecht The Netherlands; ^11^ Newcastle University, Wolfson Childhood Cancer Research Centre, Translational and Clinical Research Institute Newcastle United Kingdom

## Abstract

Anti‐CD19 CAR NK cells may provide a promising non‐HLA‐restricted immune cell product and have been clinically studied primarily on low‐grade B‐cell lymphoma patients. We used retroviral gene transfer to generate aCD19 CAR NK cells from the peripheral blood of healthy volunteers. We evaluated their efficacy in B‐lineage acute lymphoblastic leukemia (BCP‐ALL) using patient‐derived xenograft (PDX) cells in vitro and in vivo. aCD19 CAR NK cells showed potent specific cytotoxicity against eleven BCP‐ALL PDX models in vitro. When used as monotherapy in vivo, they provided a survival benefit, albeit complete remissions were not achieved. Due to the low accumulation of aCD19 CAR NK cells in the bone marrow, we used targeted pharmacotherapy based on venetoclax, dexamethasone, and dasatinib to induce remission in BCR‐ABL‐positive ALL and combined it with aCD19 NK cell therapy for consolidation. Overlapping therapy enhanced aCD19 CAR NK cell cytotoxicity in vitro and significantly prolonged survival in two high‐risk BCP‐ALL PDX models with individual long‐term remissions. Relapse cells showed no signs of therapy‐induced evolution as CD19 expression, sensitivity to venetoclax, and aCD19 CAR cell cytotoxicity remained unchanged. These data demonstrate the potential of aCD19 CAR NK cells as a component of combinatorial therapy for BCP‐ALL, which should be further evaluated in clinical trials.

## Graphical abstract

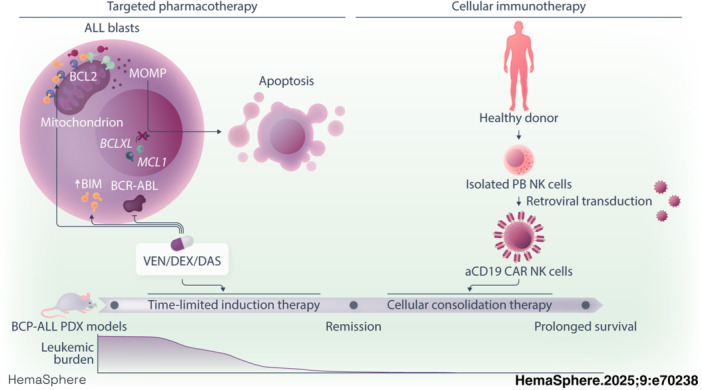

## INTRODUCTION

B‐cell precursor acute lymphoblastic leukemia (BCP‐ALL) in adults is a heterogeneous disease with a dismal prognosis in case of relapse. However, recent progress in molecular genetics has spurred risk stratification, and novel targeted therapies improve clinical outcomes. These new pharmacotherapeutic options include the CD19‐CD3 bispecific T‐cell engager blinatumomab, the CD22‐antibody–drug conjugate inotuzumab–ozogamicin (INO), and small‐molecule inhibitors such as the BCL2‐inhibitor venetoclax, as well as ABL‐targeting tyrosine kinase inhibitors (e.g., dasatinib and ponatinib) in cases of BCR‐ABL‐positive ALL.^1^, ^2^, ^3^ In addition, cellular immunotherapy with chimeric antigen receptor (CAR) T cells has recently emerged as an alternative therapeutic option for patients with relapsed BCP‐ALL. In particular, autologous T cells that express anti‐CD19 (aCD19) CARs show promising anti‐tumor activity (e.g., Tisagenlecleucel, Brexucabtagen autoleucel) in relapsed ALL patients.^4^, ^5^ Second‐generation CARs comprise an extracellular single‐chain variable fragment (scFv) recognizing a tumor antigen fused to an additional costimulatory domain upstream of the CD3ζ activation domain, such as CD28 or CD137 (4‐1BB). T cells expressing a second‐generation CAR can recognize surface target antigens independent of co‐receptor recognition to eradicate leukemic cells.^6^

However, aCD19 CAR T cell therapy can cause serious adverse events like cytokine release syndrome (CRS) by inflammatory cytokines induced during CAR T cell action and expansion, and immune effector cell‐associated neurotoxicity syndrome (ICANS). Furthermore, CAR T cell production requires autologous leukapheresis, but the quality of autologous T cells may be restricted due to preceding treatment cycles in relapsed ALL patients.^6^

In contrast to CAR T cells, CAR NK cells are not HLA‐restricted and allogeneic HLA‐mismatched aCD19 CAR NK cells do not induce CRS and ICANS.^7^ Therefore, CAR NK cells can be produced as an universal off‐the‐shelf product, which can safely be administered without needing HLA matching or risk of graft‐versus‐host disease (GvHD) induction.^8^, ^9^, ^10^, ^11^

In the first clinical study with aCD19 CAR NK cell therapy in 37 patients with CD19 + B‐cell malignancies, 48.6% of patients showed a rapid (30 days) and sustained (100 days) response, including complete remissions, and 32% of patients had a 1‐year progression‐free survival. These data demonstrate the clinical potential of aCD19 CAR NK cells in relapsed B‐cell malignancies.^12^

We hypothesized that a combinatorial approach of targeted pharmacotherapy and aCD19 CAR NK cell immunotherapy may further improve their therapeutic potential in relapsed BCP‐ALL. For this, we transduced NK cells from healthy donors with an alpha retroviral vector containing an aCD19 CAR expression cassette. We designed suitable combination therapies for BCP‐ALL patient‐derived xenograft (PDX) models in vitro. In subsequent in vivo experiments with PDX NSG mouse models, effective combination therapies significantly improved clinical outcomes.

## MATERIALS AND METHODS

### Patient material

BM and PB samples were collected from relapsed B‐lineage ALL patients (see Supporting Information S1: Table 1). Written informed consent was obtained from all patients per the Declaration of Helsinki and the Hannover Medical School Ethics Committee. Mononuclear cells were isolated using Ficoll‐Paque (Bio&Sell) density gradient centrifugation and ammonium chloride lysis of erythrocytes, and were characterized by subsequent flow cytometric analysis. Cells were either cryopreserved or directly transplanted in NSG mice for patient‐derived xenograft (PDX) generation (see below).

### Isolation, phenotypic characterization, and culture of primary human NK cells

Following the informed consent of the respective donors and in accordance with the Hannover Medical School ethics commission (#10 591_BO_K_2022), primary human untouched NK cells were purified from lymphoreduction system chambers (LRSCs) (*n* = 8) by magnetic cell separation. LRSCs from healthy donors were obtained from the Institute for Transfusion Medicine of Hannover Medical School. Using the MACSxpress® Whole Blood NK Cell Isolation Kit and the MACSxpress Erythocyte Depletion Kit (both Miltenyi Biotech), non‐target cells (e.g., T‐, B‐cells, monocytes, and erythrocytes) were removed according to the manufacturer's instructions. Cells were analyzed for specific surface markers using the Navios flow cytometer (Navios 3L10C, Software1.3; Beckman Coulter) as described in detail.^13^

After isolation and flow cytometric analysis of untouched NK cells, cultivation and expansion started with a cell concentration of 3–4 × 10^6^ CD56^+^/CD3^−^ NK cells/mL (100% purity) in NK MACS® medium composed of NK MACS Basal Medium and 1% NK MACS Supplement (both Miltenyi Biotec), complemented by 5% human serum type AB off‐the‐clot (Bio&Sell) supplemented with 500 U/mL IL‐2 (Proleukin® S; Clinigen/Novartis, Schwalbach a. Ts), 140 U/mL recombinant huIL‐15, and 10 U/mL huIL‐21 for cell activation (both Miltenyi Biotec). NK cells were expanded at a concentration of 3–4 × 10^6^ cells/mL for up to 20 days in the respective medium without huIL‐21. On Days 6–8 after isolation, cells were stained for activation markers NKp30, NKp46, and NKG2D as well as exhaustion markers PD‐1, LAG‐3, and TIM‐3 for flow cytometric analysis.

### Pharmacologic agents

For in vitro studies, dasatinib (Santa Cruz Biotechnology), venetoclax, and dexamethasone (both Selleck Chemicals) were solubilized in dimethyl sulfoxide (DMSO) or PBS to 10 mM stock, respectively, and then supplemented to the culture medium at the required concentrations.

### Ex vivo drug treatment and assessment of cytotoxicity

PDX cells were isolated from the bone marrow or spleen of end‐stage leukemic NSG mice (see below). ALL PDX cells (1 × 10^6^ cells/mL) were cocultured in flat‐bottom 96‐well plates (Thermofisher) seeded with 4 × 10^3^ MSCs per well in MSC medium (DMEM low glucose, 20% FCS, 25 mM HEPES‐buffer, 1% penicillin/streptomycin, 2 ng/mL βFGF‐2) 4 h prior to treatment in SFEM II medium (Stemcell Technologies) supplemented with 20% FCS, 20 ng/mL recombinant IL‐3, 10 ng/mL recombinant IL‐7 (both Peprotech), and 1% penicillin/streptomycin. Cells were treated with increasing concentrations of venetoclax, dexamethasone, and dasatinib alone or in combination in fixed ratios for 48 h and subsequent huCD19 antibody (anti‐CD19‐APC; BioLegend) and calcein AM/propidium iodide staining (Serva) was performed. Cells were analyzed using a flow cytometer, and data were analyzed using Cell‐Quest Pro software (BD Bioscience). Drug combination indices were calculated using GraphPad Prism (Version 7.04) and CompuSyn Software.

### Cytotoxicity assays

Specific cytotoxicity of aCD19 CAR NK cells against PDX cells (1 × 10^6^/mL) over 24 h was analyzed at different E:T ratios of 0.1:1; 0.2:1, 0.5:1, and 1:1 in a 96‐well plate. After incubation, cells were stained with anti‐CD56 antibody and calcein AM/PI and assayed by flow cytometry. For assay validation, we analyzed the bioluminescence of viable cells of one representative luciferase‐positive PDX model (ALL5, see below) as second readout using the Bright Glo luciferase assay system (Promega) in a Mithras LB940 microplate reader (Berthold Technologies).

To assess the combined killing effect of drugs and aCD19 CAR NK cells on PDX cells, the respective concentrations of the drugs (24 h) were chosen so that a cell lysis of about 20%–30% could be demonstrated. We assessed either simultaneous or sequential treatment. For this, PDX cells were treated with venetoclax/dexamethasone (VD) or venetoclax/dexamethasone/dasatinib (VDD) for 24 h and cocultured with aCD19 CAR NK cells either for the whole 24 h (simultaneous treatment) or for the last 4 h (sequential treatment). Subsequently, cells were stained for CD56 (anti‐CD56‐APC; BioLegend) and calcein AM/PI. CD56^−^/PI^−^/calcein AM^+^ cells were analyzed using a FACS Calibur flow cytometer, and data were analyzed using Cell‐Quest Pro software (BD Bioscience).

### Animal experiments

For the generation of ALL PDX in vivo models, patient samples (Supporting Information S1: Table 1) were transplanted into the tail vein of 8‐ to 10‐week‐old NSG mice irradiated with 2.5 Gy. Leukemia engraftment was monitored by immunophenotyping of peripheral blood (huCD45, huCD19). Mice were killed, and cells were isolated from the bone marrow and subsequently transduced with either Chili‐Luc (ALL4) or SLIEW (ALL6) lentiviral vector containing luciferase and RFP or GFP, respectively, as described earlier.^14^ Cells were washed and further passaged in NSG mice. Chili‐Luc and SLIEW‐transduced PDX cells were sorted for RFP or GFP expression, respectively (Sorter Facility MHH) and adoptively transplanted in NSG mice. PDX cells were isolated from the bone marrow and spleen of leukemic mice showing >95% human CD45+ /CD19+ cells, with no detection of CD3/CD14/CD90/CD105/CD56 or CD16 expression. All cells negative for human CD45 stained positive for murine CD45, demonstrating some minor contamination by murine hematopoietic cells (Supporting Information S1: Table 2). Immunophenotypes of PDX cells and the respective initial patient material remained stable and all (>99.9%) RFP‐ or GFP‐positive cells expressed CD19 (Supporting Information S1: Table 2). All cells were cryopreserved before use in in vitro and in vivo experiments and checked for viability after thawing (Supporting Information S1: Table 2).

For in vivo experiments, ALL4 and ALL6 PDX cells stably expressing luciferase (1 × 10^6^) were intravenously transplanted into the tail veins of female recipient NSG mice. Upon confirmed engraftment by IVIS bioluminescence imaging,^14^ mice were randomly allocated to each group and treated with the respective antineoplastic agents. Dexamethasone (1 mg/kg) was applied orally in 0.1 M sodium citrate by oral gavage 5 days per week in combination with venetoclax (20 mg/kg) in a vehicle consisting of 60% phosal, 30% PEG 400, and 10% ethanol with a treatment delay of minimum 2 h. The combination of DAS (10 mg/kg) and DEX was applied orally in 0.1 M sodium citrate in combination with venetoclax (20 mg/kg) with a treatment delay of a minimum of 2 h. Detailed dosing information is shown in the figure legends. Tumor burden was assessed by BLI. D‐Luciferin (1 mg/mouse) (AppliChem) was applied intraperitoneally. Life imaging of tumor growth was detected weekly using IVIS Lumina II (Perkin Elmer). Bioluminescence radiance was analyzed using Living Image 4.7.4.

After 4 weeks of oral gavage, mice were again randomized into different treatment groups and intravenously injected with 1 × 10^7^ aCD19 CAR NK or WT NK cells (control mice) once a week over 3 weeks. We used at least 3 different NK cell donors for each in vivo experiment pairing aCD19 CAR+ NK cells with WT NK cells from the respective donor as a control. For these studies, CAR+ cells ranged from 5.23% (±5.95) to 10.68% (±4.34) and lysis capacity tested on 697 cells ranged from 22.72% (±2.77) to 55.55% (±16.25). For the persistence of aCD19 CAR NK or WT NK cells, peripheral blood was analyzed 5 days after the second NK injection by flow cytometry for human CD45/CD56 double‐positive cells. A luminescence analysis was performed every week to determine the progression of ALL blasts.

To assess treatment‐related toxicity for both pharmacotherapy and cellular therapy, we determined body weight, visual appearance, and cage activity daily during treatment periods.

All animal studies were carried out in accordance with the German animal protection law and with the European Communities Council Directive 86/609/EEC and 2010/63/EU for the protection of animals used for experimental purposes. All experiments were approved by the Local Institutional Animal Care and Research Advisory Committee and permitted by the local authority, the Niedersächsisches Landesamt für Verbraucherschutz und Lebensmittelsicherheit [No. 33.14‐42502‐04‐19/3217].

### RNA sequencing

According to the manufacturer's instructions, RNA was extracted from the bone marrow or spleen of relapsed mice using the RNeasy Mini Kit (Qiagen). The quality and integrity of RNA were assessed using the Bioanalyzer (Agilent). RNA libraries were constructed using the Kapa RNA Hyperprep kit with riboerase (HMR) (Roche) according to the manufacturer's instructions. After assessing libraries by Qubit (Thermo Fischer Scientific) and TapeStation (Agilent) for quality, libraries were sequenced on the NovaSeq. 6000 (2 × 150 bp) by Illumina with paired‐end sequencing. DESeq. 2 (Version 1.44.0) was used to perform differential expression analysis on RNA count data.

### Analysis of relapsed PDX samples

At the end of the study, PDX cells were isolated from the spleen and bone marrow at the time of relapse when termination criteria were fulfilled. Cells were stained with anti‐huCD56‐APC (BioLegend), anti‐huCD19‐PE, or anti‐huCD19‐FITC (both BioLegend) according to the reporter fluorescence protein of the respective PDX model. For functional analysis, isolated cells were subjected to the respective assays. Cells were analyzed using a FACS Calibur flow cytometer, and data were analyzed using Cell‐Quest Pro software (BD Bioscience). For the assessment of apoptosis or MOMP induction by venetoclax cells, they were subjected to the respective assays (see above).

### Statistical analysis

Error bars represent SD if not otherwise stated. Statistical analysis was conducted using GraphPad Prism software (Version 7.04 and 9.5.0). Statistical significance was assessed using Student's *t*‐tests or, where appropriate, one‐ or two‐way analysis of variance (ANOVA), followed by Bonferroni‐adjusted post hoc multiple comparisons. Kaplan–Meier statistical analysis was performed using the log‐rank test. *p* < 0.05 were considered significant.

Additional methods can be found in the Supporting Information.

## RESULTS

### Primary PB‐derived human NK cells can effectively be transduced with a retroviral aCD19 CAR construct

**Figure 1:**
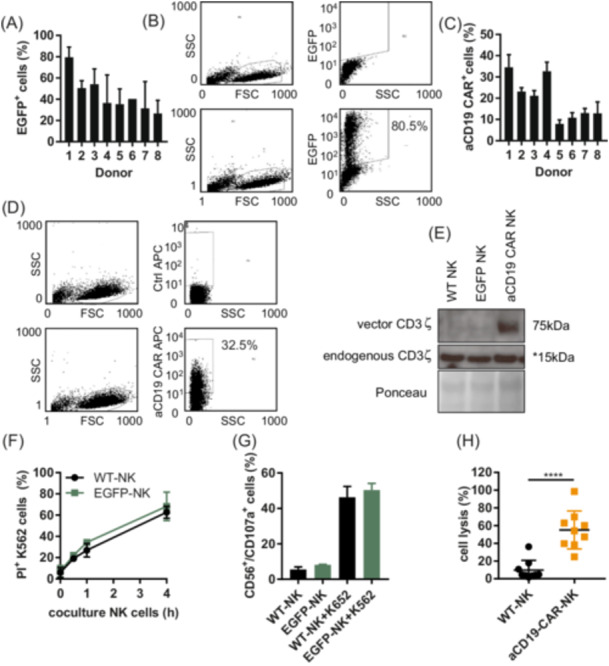
**Efficient transduction of primary human NK cells isolated from eight different donors**. **(A)** Primary human NK cells were isolated and transduced with an EGFP‐encoding alpharetroviral supernatant, and EGFP expression was analyzed using flow cytometry. The expression level on Day 4 is shown as a percentage of EGFP. Shown are the mean values ±SD from *n* = 8. **(B)** Dot plots of a representative experiment showing EGFP protein in nontransduced (upper blots) and EGFP‐transduced NK cells (lower blots) isolated from one donor. **(C)** NK cells were transduced with aCD19 CAR‐expressing alpharetroviral supernatant. Analysis of aCD19 CAR‐transduced NK cells were compared to nontransduced NK cells by flow cytometry. The expression level on Day 4 is shown as a percentage of aCD19 CAR expression. Shown are the mean values ±SD *n* = 8. (**D**) Dot plots of a representative experiment showing aCD19 CAR surface expression in NK cells (lower blots) or nontransduced NK cells (upper blots) isolated from one donor. **(E)** The aCD19 CAR‐expressing NK cells were detected by western blot analysis using the CD3ζ antibody. It also detects the endogenously expressed protein CD3ζ, marked with an asterisk (15 kDa). Ponceau S served as a loading control. Lane 1 shows wild‐type NK cells, lane 2 EGFP‐expressing cells, and lane 3 aCD19 CAR‐expressing NK cells. **(F)** NK cell cytotoxicity against K562 cells was analyzed by coculture of wild‐type or EGFP‐expressing NK cells and K562 (E:T ratio 1:1) for 1–4 h. Data show the mean values ±SD. **(G)** Degranulation assay of NK cells expressing EGFP. Extracellular CD107a/CD56 expression was analyzed by coculture of wild‐type or EGFP‐expressing NK cells and K562 cells (E:T ratio 1:1, 1 h). The graph represents the mean values ±SD. **(H)** Cytotoxic activity of wild‐type or aCD19 CAR NK‐expressing NK cells against 697 cells (E:T ratio 1:1). After 4 h of coincubation, the cytotoxicity was determined by flow cytometry. Student's *t*‐test was used to compare cytotoxic activity, ****P ≤ 0.0001.

**Figure 2:**
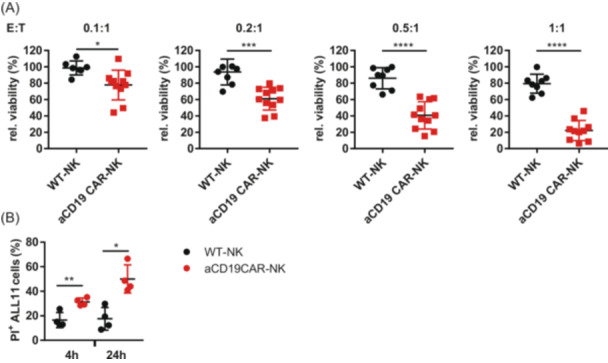
**Cytotoxic activity of primary human NK cells expressing aCD19 CAR against different BCP‐ALL PDX cells**. **(A)** Coincubation experiments of wild‐type and aCD19 CAR NK cells with BCP‐ALL PDX cells (*n* = 11) were carried out at various ratios (E:T: 0.1:1, 0.2:1, 0.5:1, and 1:1) for 24 h and the degree of cell lysis was analyzed by flow cytometry. **(B)** Different transduced aCD19 CAR NK cell donors (*n* = 4) were tested for their cell lysis against PDX cells ALL11 at a ratio of 1:1 after 4 and 24 h of coculture. Cytotoxic effects were analyzed by flow cytometry. Data show the mean values ±SD. **(A)** Student's *t*‐test or **(B)** one‐way ANOVA with the Bonferroni post hoc test were used to compare cytotoxic activity statistically. *P ≤ 0.05), **P ≤ 0.01, and ****P ≤ 0.0001.

In this study, we used alpharetroviral^15^ transduction for stable expression of aCD19‐CAR in highly purified primary human NK cells isolated from the peripheral blood of healthy donors. NK cell subset analysis (CD56^bright^CD16^−^, CD56^+^CD16^+^, and CD56^dim^CD16^−^) of the respective donors showed only marginal differences among all donors upon NK cell isolation (Supporting Information S1: Table 3). As expected, the CD56^bright^CD16^−^ subset increased about 10‐fold during in vitro expansion in the presence of hIL‐2 and hIL‐15 over six days (Supporting Information S1: Table 4). At Days 6–8 after isolation, NK cells expressed 96.66% (±1.77) NKG2D, 99.70% (±0.13) NKp30, and 99.05% (±0.27) NKp46 with varying proportions of NKp46^bright^ cells (Supporting Information S1: Figure 1). Expression of the exhaustion marker PD‐1 and LAG‐3 was low or negative (LAG‐3: 6.28% ± 3.68, PD1: 0.17% ± 0.05%), while TIM‐3 was heterogeneously expressed in 39.67% (±13.27) of NK cells (Supporting Information S1: Figure 2).

The aCD19 CAR alpharetroviral vector used for transduction encodes a codon‐optimized aCD19 CAR under the control of a MPSV promoter. A matched EGFP‐expressing vector was used as a control.^15^ The alpharetroviral vector was pseudotyped with the feline endogenous virus chimeric construct RD114‐TR.^16^ Vectofusin‐mediated transduction of eight human primary NK cell donors was performed using either EGFP‐ or aCD19 CAR alpharetroviral supernatants. The transduction efficacy was analyzed 4 days after transduction using EGFP fluorescence in controls and ranged between 26.7 and 79.5% (mean 43.7% (±16.8)) depending on individual NK cell donors (Figure 1A,B). In aCD19 CAR NK cells, CAR surface expression was analyzed by antibody staining of the aCD19 CAR region and flow cytometry (Figure 1C,D). aCD19 CAR expression ranged from 7.9% to 34.5% (mean 19.5% (±10.1)), again depending on individual NK cell donors. Expression of aCD19 CAR was also analyzed by immunoblot to detect CD3ζ (fused with the CAR vector) at 75 kDa. The lower signal (approximately 15 kDa) results from endogenously expressed CD3ζ in NK cells (Figure 1E). In terms of NK cell function, we did not observe differences in cytotoxicity or degranulation potential against K562 cells between nontransduced wild‐type (WT) NK cells or EFGP transduced NK cells (EGFP‐NK cells) in 4‐h cocultures with an effector:target cell ratio (E:T ratio) of 1:1 (Figure 1F,G). The specific CD19‐mediated cytotoxicity of aCD19 CAR‐NK cells was determined by a 4‐h coculture with 697 cells in an E:T ratio of 1:1. Cell lysis was assessed by propidium iodide staining and ranged from 24.7% to 98.5% (mean 55.1% (±21.5), depending on different NK cell donors (Figure 1H). In contrast, WT NK cells induced a mean cell lysis of only 9.7% (±11.1).

### Primary aCD19 CAR NK cells are highly effective against human BCP‐ALL PDX cells in vitro

**Figure 3:**
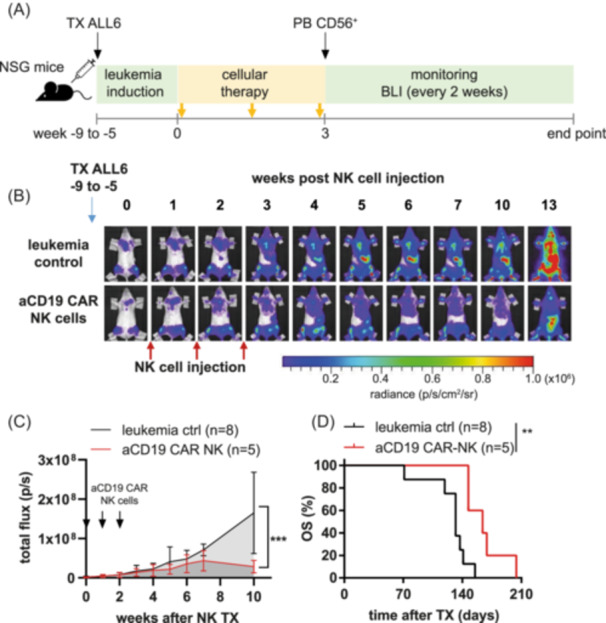
**aCD19 CAR NK cell therapy dampens leukemia progression, with a mild survival benefit. (A)** Treatment scheme of an in vivo PDX experiment with ALL6. **(B)** ALL6 BCP‐ALL PDX cells were intravenously transplanted in the tail vein of NSG mice. Tumor monitoring was performed by in vivo bioluminescence imaging (BLI) every week. After confirmed engraftment, mice were randomly allocated to the control or treatment group. The treatment group received injections of 1 × 10^7^ aCD19 CAR NK cells i.v. over 3 consecutive weeks. During the treatment, mice received 2500 U IL‐15 intraperitoneal thrice weekly to improve NK cell persistence in vivo. **(C)** BLI signals were quantified by Living Image 4.7.4 software. ***P < 0.001 two‐way ANOVA. **(D)** Kaplan–Meier survival curve of ALL6 untreated leukemia control mice and aCD19 CAR NK cell‐treated mice. **P < 0.01 log‐rank test.

Dose‐dependent cytotoxicity of aCD19 CAR NK cells was investigated in BCP‐ALL PDX cells with various genetic aberrations (*n* = 11; for detailed information, see Supporting Information S1: Table 2) at different E:T ratios (1:1, 0.5:1, 0.2:1, and 0.1:1). PDX cells were cultured on a mesenchymal stromal cell (MSC) feeder cell layer, and cytotoxicity was analyzed after 24 h by flow cytometry or by a luciferase activity assay. BCP‐ALL PDX cells were effectively killed by aCD19 CAR NK cells even at E:T ratios below 1:1 (Figure 2A and Supporting Information S1: Figure 3A,B). In contrast, the corresponding WT NK cells showed almost no cytotoxicity against the PDX cells. Significant anti‐leukemic activity was already detectable after 4 h of coculture in BCP‐ALL PDX cells (Figure 2B).

### Induction therapy with primary aCD19 CAR NK cells prolongs survival in a BCP‐ALL PDX model in vivo

**Figure 4:**
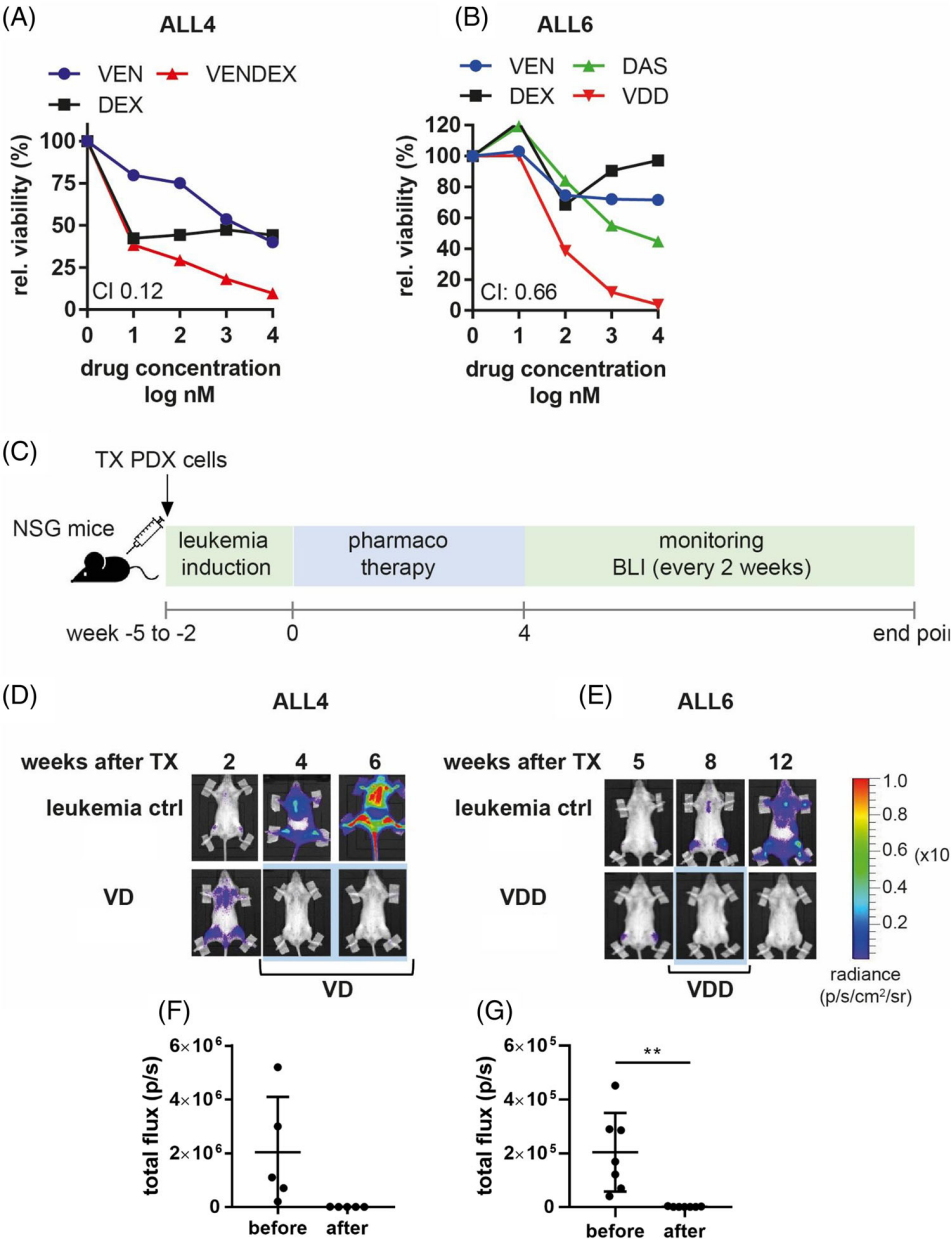
**VEN/DEX and VEN/DEX/DAS are effective induction therapies for BCP‐ALL.** ALL4 **(A)** and ALL6 **(B)** PDX cells were cocultured on MSC and treated for 48 h with VEN, DEX, and DAS or as a combination of VEN/DEX (VD) **(A)** or VEN/DEX/DAS (VDD) **(B)** in increasing concentrations. Cell viability was assessed by CD19/calcein AM and PI staining via flow cytometry. Combination indices were calculated using CompuSyn software. CI < 1 indicates synergistic cytotoxicity. **(C)** Treatment scheme of ALL PDX in vivo experiments. ALL4 **(D**, **F)** or ALL6 **(E**, **G)** cells were transplanted in the tail vein of NSG mice. After BLI‐assessed confirmed engraftment, mice were treated orally with VEN/DEX (VD) or VEN/DEX/DAS (VDD), respectively, 5 times a week for 4 weeks. BLI was performed to monitor leukemic burden. **(F**, **G)** Quantification of BLI (total flux) on the day of start of treatment and after 4 weeks.

To determine the therapeutic potential of primary aCD19 CAR NK cells as induction therapy in vivo, the luciferase‐expressing BCP‐ALL PDX model ALL6 (1 × 10^6^ cells) was transplanted via the tail veins of NSG mice. Engraftment was confirmed by in vivo bioluminescence imaging (BLI) (total flux min. 1 × 10^5^ p/s) between 5 and 9 weeks after transplantation. Mice were randomly allocated to the untreated leukemia control group or treated intravenously with 1 × 10^7^ aCD19 CAR NK cells once a week for 3 consecutive weeks (see the treatment scheme Figure 3A). 2500 U hIL‐15 were applied i.p. three times a week to improve NK cell persistence. To assess the side effects of NK cell therapy, body weight and a clinical score were determined for all mice. The body weight remained stable in all mice during the treatment period, and no clinical signs of toxicity were observed (Supporting Information S1: Figure 4A). Although a mild delay in leukemia progression was achieved by aCD19 CAR NK cell treatment, therapy did not eradicate leukemia (Figure 3B,C). However, aCD19 CAR NK cell treatment significantly prolonged survival, as shown in Figure 3D (median survival 132 vs. 164 days). There was no difference in the proportion of CD19+ cells in the bone marrow between the leukemia control and aCD19 CAR‐NK cell‐treated mice (Supporting Information S1: Figure 4B). The number of aCD19 CAR NK cells 4–5 days post second NK cell application in the peripheral blood was 7.98% (±6.39). At the same time, 4.73% (±3.96) huCD45+/CD56− (PDX) cells were detectable, which corresponds to a numeric E:T ratio of ≤2:1 in peripheral blood (Supporting Information S1: Figure 4C). Since the main leukemic burden at this stage of disease resides in the bone marrow, we assessed the homing of NK cells to the bone marrow 4–5 days after the second WT NK cell injection in NSG mice. While NK cells are easily detectable in PB (15.52% (±14.07)) and spleen (12.54% (±9.69)), the proportion of NK cells in the bone marrow was marginal (0.40% (±0.23)) (Supporting Information S1: Figure 4D). In total 5 × 10^4^ to 1.6 × 10^5^ CD56+ cells could be isolated from the bone marrow of both hind limbs, whereas the NK cell count isolated from the spleen was significantly higher (Supporting Information S1: Figure 4E). To improve aCD19 CAR NK cell therapy, we analyzed combinatorial approaches with pharmaco‐based induction therapy.

### Venetoclax‐containing combination treatment is effective as induction therapy in vivo

As shown previously,^17^, ^18^ BCP‐ALL PDX cells are susceptible to combination therapy with BH3 mimetics such as venetoclax. Here, we used a combination of venetoclax (VEN) and dexamethasone (DEX) for BCR‐ABL‐negative ALL and VEN/DEX + dasatinib (DAS) for BCR‐ABL‐positive ALL. These combinations synergistically induced cell death in two PDX models in vitro, with CI values of 0.12 and 0.66 (values <1 considered synergistic), respectively (Figure 4A,B). Next, VEN/DEX and VEN/DEX/DAS were tested in vivo as induction therapy in these BCP‐ALL PDX models. After BLI‐confirmed leukemic engraftment, mice were treated for 4 weeks with either VEN/DEX or VEN/DEX/DAS (see the treatment scheme in Figure 4C). Both regimens induced complete remissions in mice (Figure 4D–G), as assessed by BLI total flux <2 × 10^4^ p/s, and could, therefore, be used as induction therapy in a combinatorial pharmacotherapy approach with subsequent cellular therapy with aCD19 CAR NK cells.

### Venetoclax‐based pharmacotherapy regimens augment cytotoxicity of aCD19 CAR NK cell therapy in vitro

**Figure 5:**
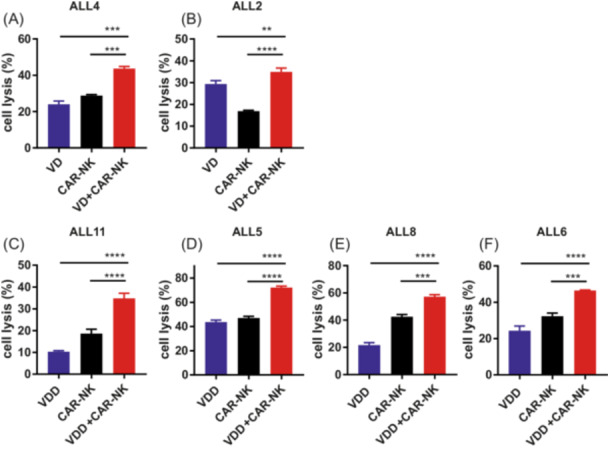
**Enhanced cytotoxicity of a combination of pharmacotherapy and cellular aCD19 CAR NK cell therapy in vitro.** BCR‐ABL‐negative ALL **(A**, **B)** or BCR‐ABL‐positive ALL **(C**–**F)** PDX cells were treated with IC20‐40 of VEN/DEX (VD) **(A**, **B)** or VEN/DEX/DAS (VDD) **(C**–**F)** combination therapy. After 20 h of pretreatment, the cells were mixed with aCD19 CAR NK cells and incubated for 4 h. Cytotoxicity was assessed using flow cytometric analysis of CD56/calcein AM/PI staining. Statistical significance was determined by one‐way ANOVA with the Bonferroni post hoc test. **P < 0.01, ***P < 0.001, and ****P < 0.0001. ANOVA, analysis of variance.

Previous reports suggested that BH3 mimetics could enhance WT NK cell‐mediated killing of leukemic cells,^19^, ^20^ while the effects of dexamethasone and dasatinib on NK effector cell function have shown conflicting outcomes.^21^, ^22^, ^23^, ^24^ Therefore, we analyzed the co‐treatment of CD19‐positive BCP‐ALL PDX cells (*n* = 6) with venetoclax‐based pharmacotherapy (VEN/DEX or VEN/DEX/DAS) for 24 h, either with simultaneous or sequential (final 4 h of incubation) addition of aCD19 CAR NK cells to the cell culture. Pharmacologic concentrations were chosen individually for each PDX model to kill equal amounts of the respective PDX cells (Supporting Information S1: Table 5). Simultaneous treatment with pharmacotherapy and aCD19 CAR NK cells enhanced cytotoxicity significantly compared to pharmacotherapy alone (Supporting Information S1: Figure 5). Addition of aCD19 CAR NK cells for 4 h to pretreated BCP‐ALL PDX cells enhanced cytotoxicity 1.8–3.5‐fold in two BCR‐ABL‐negative models (Figure 5A,B) and in four BCR‐ABL‐positive ALL PDX models (Figure 5C–F) in vitro.

### Venetoclax‐based induction with subsequent aCD19 CAR NK treatment achieves long‐term remission

**Figure 6:**
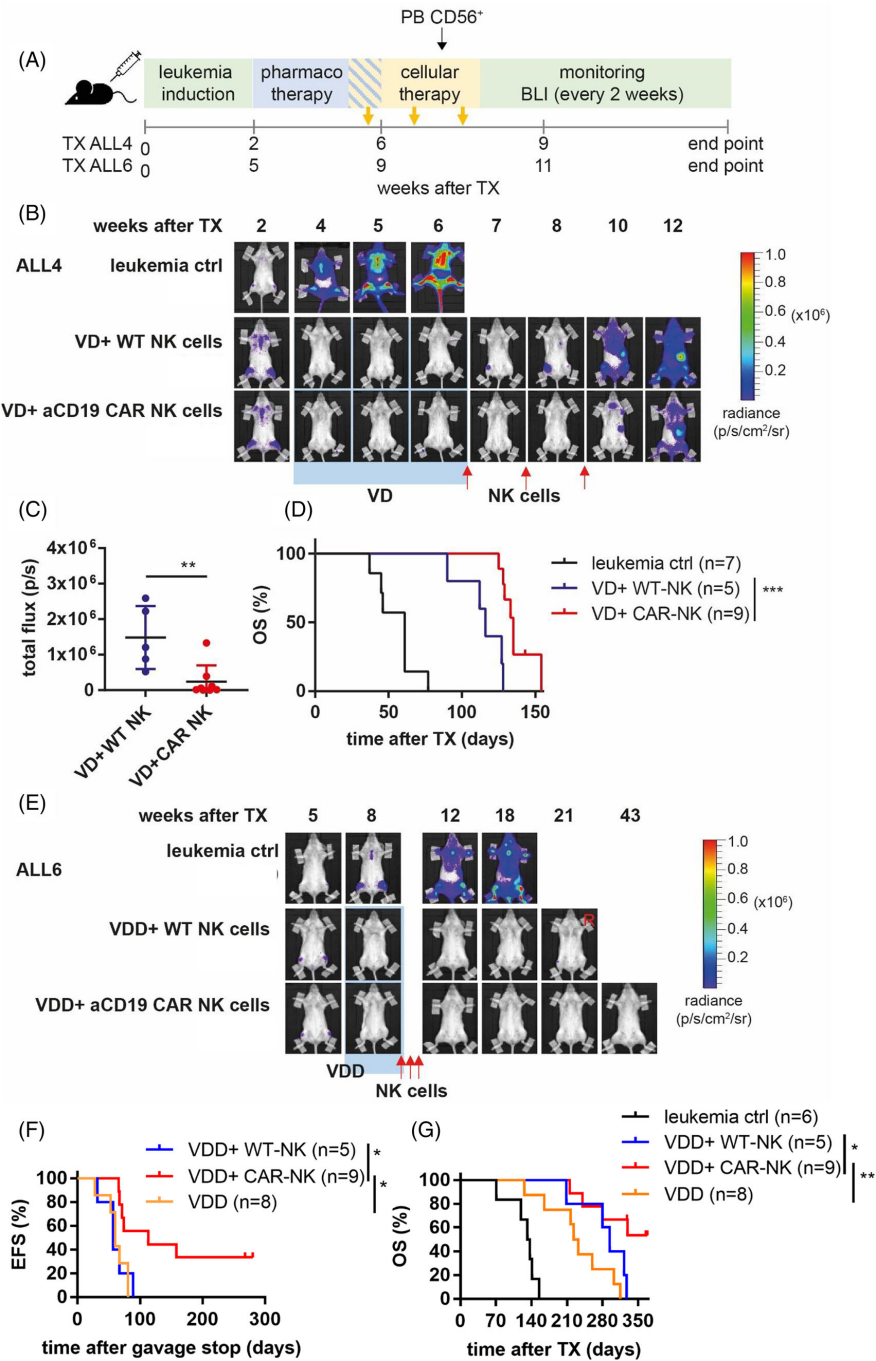
**Sequential combination of venetoclax‐based pharmacotherapy and aCD19 CAR NK cell immunotherapy induced complete remissions and prolonged survival in different PDX models in vivo.** (**A**) Schematic treatment schedule. **(B**–**G)** ALL4 cells **(B**–**D)** or ALL6 cells **(E**–**G)** were systemically engrafted in NSG mice. After confirmed engraftment (by BLI), mice were randomly allocated to an untreated leukemia control group or treated for 4 weeks orally with **(B**–**D)** venetoclax (20 mg/kg) and dexamethasone (1 mg/kg) (VD) 5 days a week in case of ALL4 and **(E**–**G)** venetoclax (20 mg/kg), dexamethasone (1 mg/kg), and dasatinib (10 mg/kg) (VDD) 5 days a week in case of ALL6. In Week 4 of pharmaco‐treatment, mice were again randomly allocated either to the WT NK cell or the aCD19 CAR NK cell treatment group. Cellular treatment started in Week 4 of pharmacotherapy by an i.v. application of 1 × 10^7^ cells once a week for 3 consecutive weeks. During this time, mice received 2500 U IL‐15 i.p. 3 times a week. **(B**, **E)** Representative BLI for one mouse per treatment group. **(C)** Quantification of BLI (total flux) week 10 after TX **(F)** event‐free survival (EFS) of ALL6 mice as assessed by BLI. **(D**, **G)** Kaplan–Meier overall survival (OS) curve of ALL4 **(D)** and ALL6 **(G)** mice. **(C)** *P < 0.05 Students *t*‐test. *P < 0.05 and ***P < 0.001 log‐rank test.

Based on these in vitro data, we tested this combinatorial approach with pharmacotherapy for remission induction and aCD19 CAR NK cells for consolidation with one week of concurrent treatment in vivo, as outlined in Figure 6A.

NSG mice were intravenously transplanted with two high‐risk BCP‐ALL PDX models (1 × 10^6^ cells). PDX model ALL4 with a KMT2A::MLLT1 fusion was established from a patient who had relapsed after allogeneic stem cell transplantation. PDX model ALL6 was established from a BCR‐ABL‐positive lymphatic blast crisis with a complex karyotype after allogeneic stem cell transplantation (Supporting Information S1: Table 1).

Following engraftment, as confirmed by BLI (total flux min. 1 × 10^5^ p/s), ALL4 and ALL6 NSG mice were treated with VEN 20 mg/kg and DEX 1 mg/kg, or VEN 20 mg/kg, DEX 1 mg/kg, and DAS 10 mg/kg, respectively, for 4 weeks. In Week 4 of induction therapy, treatment with aCD19 CAR NK cells or WT NK cells was applied with concurrent pharmaco‐ and immune cell therapy for the last week. Cellular therapy was applied once a week for 3 consecutive weeks in total (Figure 6A). During this time, the mice received IL‐15 2500 U/mice three times a week for NK cell boosting. The number of NK cells in the PB was assessed 5 days after the second NK cell application (Supporting Information S1: Figure 6A,B). No abnormal symptoms or clinical signs of toxicity were observed, except for moderate weight loss, most probably due to oral drug application by gavage in almost all mice. However, all mice regained weight after the end of oral drug application. In contrast to pharmacotherapy, the injection of CAR or WT NK cells had no significant clinical side effects and neither reduced nor delayed body weight recovery (Supporting Information S1: Figure 6C–E).

In both PDX models, induction pharmacotherapy induced complete remission in all mice, as assessed by BLI. Although remissions in ALL4 mice were short (median 18 days), mice treated with VD + aCD19 CAR NK cells had a significantly lower leukemic burden on Day 20 after the first NK cell application compared to VD + WT NK cell‐treated mice (Figure 6B,C). In addition, the median overall survival increased significantly from 74 days (VD + WT NK cells) to 96 days (VD + aCD19 CAR NK cells) (Figure 6D).

For NSG mice engrafted with ALL6, event‐free survival (EFS) was significantly longer upon VDD + aCD19 CAR NK cell treatment (mean 113 days) as compared to VDD + WT NK cell treatment (mean 57 days) or pharmacotherapy alone (VDD) (mean 60 days) (Figure 6F). Accordingly, the overall survival did not differ significantly between the VDD and VDD + WT NK cell‐treated mice.

In contrast, VDD + aCD19 CAR NK cell treatment led to long‐term leukemia‐free survival (>250 days) in 3 out of 9 mice (33%) (Figure 6E,F). Overall survival was also significantly improved in VDD + aCD19 CAR NK cell‐treated mice, where 5 mice (out of 9, 55.6%) survived the whole observation period of 1 year, while mice treated with no NK cells or WT NK cells showed a median overall survival of 227.5 days and 294 days, respectively (Figure 6G).

### Consolidation treatment with aCD19 CAR NK cells does not induce phenotypic alterations in relapsed ALL cells

**Figure 7:**
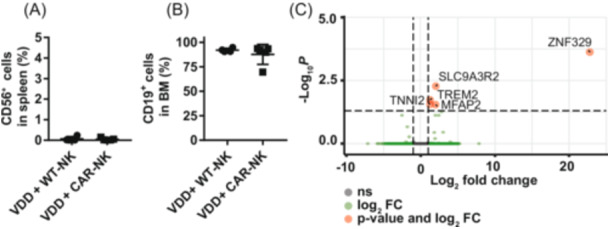
**Analysis of relapses from ALL6 NSG mice treated with pharmacotherapy and cellular immunotherapy**. **(A**, **B)** Flow cytometry analyses of CD56^+^ cells in the spleen and CD19^+^ cells of total nucleated viable cells in the bone marrow of relapsed ALL6 mice (*n* = 5). **(C)** Volcano plot of mRNA expression between VENDEXDAS (VDD) + aCD19 CAR NK treatment vs. VENDEXDAS (VDD) + WT NK treatment (*n* = 3). The *x*‐axis indicates the log_2_ fold change between conditions and the *y*‐axis indicates the negative logarithm of the adjusted *P* value. Genes indicated in red have a *P* > 0.5 and a log_2_ fold change >2. Total = 58,804 variables. mRNA, messenger RNA.

To study the potential factors involved in the selection of resistant phenotypes, we first analyzed the presence of NK cells and CD19 and gene expression signatures in ALL6 leukemic cells at the time of relapse when termination criteria were fulfilled (Figure 7A–C; for exact time points, see Supporting Information S1: Table 6). Splenocytes were analyzed for CD56 (Figure 7A) and bone marrow cells for CD19 expression (Figure 7B) by flow cytometry. No CD56^+^ NK cells were detectable in the spleen of relapsed mice. In the bone marrow, high infiltration by CD19‐positive cells was detected (more than 90% in all but one mouse). Phenotypical analysis of all relapsed ALL4 mice revealed considerable homogeneity and almost identical results, with no detection of CD56^+^ cells in the spleen and >92% infiltration of CD19^+^ cells in the bone marrow (Supporting Information S1: Figure 7A,B).

Furthermore, transcriptome analysis of ALL6 cells of relapsed mice with aCD19 CAR versus WT NK cell consolidation treatment by RNA sequencing revealed differential regulation of only five genes (ZNF329, SLC9A3R2, TREM2, TNNI2, and MFAP2), with no obvious functional annotation (Figure 7C). To evaluate if these marginal transcriptomic changes resulted in resistance, we further analyzed relapsed cells for functional apoptosis induction either by aCD19 CAR NK cells or by short‐term VEN treatment in vitro.

### Relapsed cells remain responsive to aCD19 CAR NK cell cytolytic activity and to venetoclax in vitro

**Figure 8:**
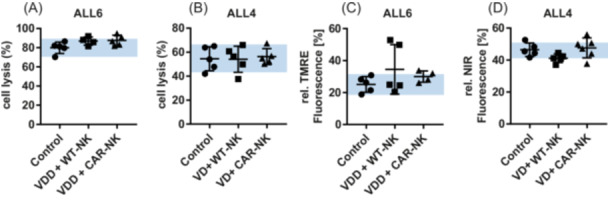
**Functional analysis of relapses from ALL4 and ALL6 NSG mice treated with pharmacotherapy and cellular immunotherapy.** Functional analysis of the relapsed cells concerning **(A**, **B)** cell lysis induced by 24 h in vitro treatment with aCD19 CAR NK cells and **(C**, **D)** VEN‐mediated MOMP induction after 3 h in comparison to PDX cells isolated from untreated mice (control). The blue area indicates the 95% confidence interval of the control cells.

To functionally characterize relapsed cells, in vitro sensitivity toward aCD19 CAR NK cells and VEN‐mediated mitochondrial outer membrane permeabilization (MOMP) were analyzed (Figure 8). CAR NK cell‐induced killing of relapsed cells (E:T ratio of 1:1) remained very effective (between 82% and 95% for ALL6 and between 38% and 67% for ALL4) and was comparable to the respective cells from untreated leukemic mice (control) (Figure 8A,B). Finally, 3 h of treatment of relapsed cells with 1 µM VEN still induced MOMP (as assessed via a decrease in TMRE‐fluorescence for ALL6 cells to 20%–53% and a decrease in NIR‐fluorescence for ALL4 cells to 37%–56%), with no difference to cells from the respective control mice (Figure 8C,D). These data indicate that both aCD19 NK cells and venetoclax can still effectively trigger apoptosis without selection of resistance phenotypes in relapsed cells.

## DISCUSSION

CAR NK cells may provide a non‐HLA‐restricted off‐the‐shelf product for cellular immunotherapy with potentially promising therapeutic perspectives. Accordingly, Marin et al. recently reported on the first clinical trial using HLA‐mismatched cord blood‐derived aCD19 CAR NK cells in patients with B cell malignancies.^12^ In preliminary studies, they found a significant impact of quality criteria for individual cord blood units on aCD19 CAR NK effector function. In the phase 1 and 2 clinical trials, 8 out of 11 patients with B‐cell lymphoma showed a response, with a trend toward better responses in low‐grade lymphoma patients. Based on these data, we generated, characterized, and evaluated aCD19 CAR NK cells from the peripheral blood of healthy volunteers for therapy of BCP‐ALL.^7^ We used BCP‐ALL PDX cells and murine xenotransplantation models for preclinical in vitro and in vivo analysis, respectively.

Upon retroviral transduction, aCD19 CAR expression was heterogeneous and ranged from 7.9% to 34.5% in NK cell preparations from individual donors. In coculture experiments with K562 cells, there was no difference in degranulation and cytotoxicity between WT and transduced NK cells. In contrast, aCD19CAR NK cell‐mediated lysis of 697 cells (55.1% ± 21.1%) was much more effective compared to WT NK cell‐induced cell lysis (9.7% ± 11.1%). In coculture with BCP‐ALL PDX cells, aCD19 CAR cells, but not WT NK cells, induced rapid, strong, and dose‐dependent cytotoxicity in all BCP‐ALL PDX models tested. These data demonstrate that peripheral blood‐derived NK cells can retrovirally be transduced to obtain highly functional aCD19 CAR NK cells. NK cell expansion with IL‐2 and IL‐15 stimulation in a feeder‐free manner leads to high numbers of NKG2D^+^/NKp46^+^/NKp30^+^ NK cells, with almost no detectable expression of exhaustion markers LAG‐3 and PD‐1. Expression of TIM‐3 was detected in a mean 39% of NK cells, which is in line with earlier reports showing upregulation of TIM‐3 upon cytokine stimulation and cultivation in vitro with IL‐2, IL‐15, or IL‐12, without functional impairment with respect to degranulation potential or cytokine production.^25^

In murine BCP‐ALL PDX xenotransplantation models, monotherapy with three weekly dosages of aCD19 CAR NK cells failed to induce remission but still retarded leukemia progression and significantly prolonged overall survival. After the second NK cell application, NK cells were detected in peripheral blood and spleen, but were found at very low levels in the bone marrow.

The low presence of aCD19 CAR NK cells in the bone marrow may hamper therapeutic efficacy in BCP‐ALL with high tumor load and tumor cell proliferation in the bone marrow. In accordance with our findings, NK cell homing to the bone marrow was very low in previous studies.^26^, ^27^ Furthermore, the NK92 cell line transduced with a CD123 CAR could not be substantially detected in the bone marrow.^28^ However, the rates of NK cell migration into and out of the bone marrow are not precisely known. NK cell trafficking and prevalence in the bone marrow may be sufficient to induce antileukemic effects, with therapeutic efficacy inversely correlated with tumor load in the bone marrow, as indicated by our data. NK cell‐mediated cytotoxicity in the bone marrow may also be improved by engineering aCD19 CAR NK cells to express cofactors to enhance bone marrow homing, such as CXCR4.^26^, ^27^, ^29^, ^30^ Finally, disregarding any species differences between mice and humans, the different bone marrow involvement and tumor cell proliferation may also contribute to different clinical responses to aCD19 CAR NK cell monotherapy in B‐cell lymphoma, as reported by Liu et al.^7^, ^12^ and in our preclinical BCP‐ALL models. Furthermore, our findings that aCD19 CAR NK cells can lead to a significant survival benefit in a BCP‐ALL PDX model with well‐established leukemia 5–9 weeks after cell transplantation clearly reach beyond previous preclinical in vivo studies. These studies used aCD19 CAR NK cells in xenotransplantation models with cell lines at time points Day 0–Day 3 after leukemia transplantation, showing delayed leukemia engraftment and, in some cases, slight survival benefits.^31^, ^32^, ^33^, ^34^, ^35^, ^36^

Based on the failure to induce ALL remission by aCD19 CAR NK cell monotherapy, we designed combinations of targeted pharmacotherapy with cellular immunotherapy. We first analyzed drug–aCD19 CAR NK cell combinations in vitro to detect potential adverse effects of, for example, steroids on NK cell function. Based on our previous results,^17^, ^18^, ^37^ we first confirmed induction of complete remission by venetoclax and dexamethasone as well as venetoclax, dexamethasone, and dasatinib for BCR‐ABL‐positive ALL in our murine PDX mouse models. In vitro, these pharmacotherapies enhance aCD19 CAR NK cell cytotoxicity in all six BCP‐ALL models tested. These data support the feasibility and efficacy of combinatorial pharmacotherapy and immune cell therapy, at least in vitro.

To analyze clinically relevant endpoints, we analyzed combinatorial treatment of pharmacotherapy for remission induction and aCD19 CAR NK cell therapy for consolidation in BCP‐ALL PDX murine xenografts. As inter‐donor variability in immune effector function may have a therapeutic impact, we used at least 3 different NK cell donors for each in vivo model, pairing aCD19 CAR NK cells with WT NK cells from the respective donor as controls. Our combinatorial approach prolonged survival substantially and even induced long‐term leukemia‐free survival in 33% of BCR‐ABL‐positive ALL mice. These data confirm the therapeutic potential of aCD19 CAR NK cells and demonstrate their effectiveness in mice with low leukemic burden. Within clinical protocols, aCD19 CAR NK cell therapy might be helpful as consolidation or maintenance therapy for treating BCP‐ALL patients.

Based on body weight and clinical scoring to address cytokine release syndrome, among others, we did not observe any NK cell‐related toxicity, either for NK cell therapy alone or in combination with pharmacotherapy. Indeed, weight losses during pharmacotherapy, which are most likely due to drug application by gavage, were completely compensated during subsequent NK cell therapy. Although we did not measure serum cytokine levels, several studies did not observe CAR NK‐related toxicities or prolonged elevated cytokine serum levels of, for example, IFNy, TNFa,^35^ IL6, IL2, and IL4.^28^

However, leukemic relapses still occur, and another point to consider is that combination therapies may influence the phenotype of relapsed cells. Therefore, we combined pharmacotherapy for apoptosis induction at the mitochondrion level with aCD19 CAR NK cellular therapy known to trigger apoptosis, for example, by cytolytic enzymes such as perforin and granzymes, Fas–FasL interaction, and release of cytokines. This non‐overlapping targeting for apoptosis induction may hamper the selection of resistance. Accordingly, and in line with observations made by Marin et al.,^12^ cells at relapse did not show loss of CD19 antigen expression. Furthermore, relapsed cells were still responsive to aCD19 CAR NK cell action and mitochondrial outer membrane permeabilization (MOMP) induction by venetoclax in vitro. Consequently, RNA sequencing analysis did not hint at any major and persistent transcriptomic changes in relapses of VEN/DEX/DAS + WT NK cells versus VEN/DEX/DAS + aCD19 CAR NK cell‐treated mice. This may be explained at least in part by the short half‐life and the presence of NK cells in PDX‐NSG mice as compared to the late time point of analysis at relapse with >200 days between NK cell injection and gene expression profiling.

In addition, aCD19 CAR NK cell therapy may not induce persistent gene expression alterations to select for resistance against NK cell cytotoxicity in particular, since NK cell consolidation was applied at time points with low tumor burden. The data on relapsed BCP‐ALL cells, however, indicate the potential to optimize pharmacokinetic aspects of NK cell therapy to improve clinical outcome at least in preclinical PDX‐NSG models.

In terms of mechanisms leading to BCP‐ALL relapses, we did not observe any functional resistance in relapsed cells, either against drugs used during induction therapy or against aCD19 CAR NK cell cytotoxicity. These data may indicate that relapses at least in these models may not be due to Darwinian selection of drug‐resistant (sub‐)clones but due to some kind of protection of tumor cells during therapy (e.g., in some kind of “niches”) and subsequent expansion.

Based on the data presented, further optimization of NK cell therapy for BCP‐ALL can be addressed. To prolong in vivo persistence and bone marrow homing of CAR NK cells, several changes in CAR vector architecture can be imagined such as enhanced NK cell survival, for example, by integration of an IL‐15 cassette.^12^ Also, preactivation of NK cells with cytokine cocktails inducing an NK memory‐like phenotype could further enhance in vivo lifespan and antitumor efficacy.^38^ Finally, different drug combinations and application schedules for both drug and NK cell therapy may be tested in the future.

In summary, highly effective aCD19 CAR NK cells can be generated from PB‐derived NK cells from healthy volunteers. In preclinical murine high‐risk BCP‐ALL PDX models, these aCD19 CAR NK cells are more effective as consolidation than induction therapy, probably linked to low NK cell prevalence in the bone marrow. Therefore, combining targeted pharmacotherapy for remission induction with aCD19 CAR NK cell therapy for consolidation or maintenance therapy may present promising therapeutic options for ALL treatments. Such combination approaches may be further optimized for relapse prevention and evaluated in future clinical trials.

## AUTHOR CONTRIBUTIONS

**Hanna Kirchhoff**: Conceptualization; investigation; writing—original draft; methodology; validation; visualization; formal analysis; funding acquisition. **Caroline Schoenherr**: Investigation; writing—review and editing; methodology; formal analysis; validation. **Lisa Fleischer**: Investigation; methodology; writing—review and editing. **Elizabeth K. Schweighart**: Investigation; writing—review and editing; methodology. **Ruth Esser**: Investigation; writing—review and editing. **Steven R. Talbot**: Writing—review and editing; formal analysis. **Axel Schambach**: Resources; writing—review and editing. **Ulrike Koehl**: Writing—review and editing; funding acquisition; resources. **Olaf Heidenreich**: Writing—review and editing; resources. **Matthias Eder**: Writing—original draft; resources; funding acquisition; conceptualization; supervision. **Michaela Scherr**: Conceptualization; investigation; funding acquisition; writing—original draft; methodology; project administration; supervision.

## CONFLICT OF INTEREST STATEMENT

The authors declare no conflicts of interest.

## FUNDING

This work was supported by Jose Carreras Leukemia‐Stiftung and H.W. & J. Hector‐Stiftung. Open Access funding enabled and organized by Projekt DEAL.

## Supporting information

Supporting information.

Supporting information.

## Data Availability

The data that support the findings of this study are available in the supplementary material of this article.
