# Supplementary material for: Combination of targeted pharmacotherapy and immunotherapy with anti‐CD19 CAR NK cells in acute lymphoblastic leukemia
Source: Hemasphere. 2025 Oct 15;9(10):e70238. doi: 10.1002/hem3.70238 (PMC12527222; doi:10.1002/hem3.70238)
Supplement: Supplementary file 1 — Supporting information. [file HEM3-9-e70238-s002.docx]

**Supplement Material and Methods**

**Cell Culture**

Cell lines 697, BV173, and K562 (DSMZ, Braunschweig, Germany) were cultured in RPMI 1640 supplemented with 10% FCS and 1% penicillin/streptomycin. 293T cells (DSMZ) were cultured in DMEM supplemented with 10% FCS and 1% penicillin/streptomycin.

**Isolation and culture of human MSCs from bone marrow**

Ethical approval was obtained from the ethical committee of Hannover Medical School for studies involving human bone marrow. Samples were collected in accordance with the Declaration of Helsinki after written informed consent of the respective donors. Isolation and culture of human MSCs were performed as described earlier (39).

**Production of retroviral supernatants and transduction of primary human NK cells**

Recombinant alpharetroviral supernatants were produced by calcium phosphate transfection of 293T cells using a RD114/TR envelope plasmid (40) (kindly provided by Dr. F.L. Cosset, Lyon, France), a codon-optimized alpharetroviral gag/pol packaging plasmid, and a alpharetroviral SIN vector plasmid containing either a EGFP marker gene or a codon-optimized third-generation anti-CD19 CAR transgene containing a signal sequence derived from granulocyte-macrophage colony-stimulting factor receptor alpha-chain, along with an anti-CD19 single-chain variable fragment derived from a mouse hybridoma FMC63 (CD19 scFv), CD28, CD137 (4-1BB), and a CD3ζ or a EGFP marker gene as described by Suerth et al. (41). Alpharetroviral supernatants were harvested 48 and 72 hours after transfection, filtered through a 0.45 µm filter (Millipore, Schwalbach, Germany), concentrated at 10.000 x g for 3 hours at 10°C and stored at -80°C.

Retroviral transductions of primary human NK cells were performed after 7 and 9 days of expansion. 2x10^5^ cells/well were transduced in 96-well plates in the presence of Vectofusin® at a final concentration of 10 µg/ml (Miltenyi Biotech) and different volumes of viral supernatants by spinoculation (90 min, 2.500 rpm, 32°C). After 24 hours, cells were maintained with fresh NK MACS® medium. Transduction efficiency was assessed 4 days after transduction directly by flow cytometric analysis of anti-CD19CAR NK- or EGFP-expression (control cells) and indirectly by assessment of specific cell lysis capacity of anti-CD19 CAR NK cells against 697 cells with effector to target ratio (E:T) of 1:1 for 4 hours by flow cytometry. Anti-CD19 CAR was detected using the CD19 CAR detection reagent (Miltenyi Biotech), including a biotinylated anti-mouse-antibody and an APC-conjugated Streptavidin (BD Bioscience).

**CD107a degranulation Assay**

WT NK cells or EGFP NK cells (2×10^5^/well) were incubated with an equal number of K562 cells in a total volume of 200 μl in 96-well plates in the presence of CD107a-APC antibody (Biolegend, Germany) for 1 hour at 37°C. The cells were washed, incubated with anti-CD56-PE labeled antibody (BD Bioscience) for 15 min, washed, and subsequently analyzed by flow cytometry. NK cells incubated with medium alone served as control.

**MOMP induction**

PDX cells of relapsed mice were treated for three hours with 1µM venetoclax at a cell density of 1x10^6^ cells/ml. Mitochondrial outer membrane permeabilization (MOMP) was assessed as described earlier (17). In brief, cells were stained with 50 nM of the mitochondrial dye TMRE (tetramethylrhodamine ethyl ester) (Sigma) or NIR (near infra-red mitochondrial membrane potential assay kit, Abcam) according to the reporter fluorescence protein of the respective PDX model for 20 minutes at 37°C. 5 µM FCCP (Carbonyl cyanide-4-(trifluoromethoxy)-phenylhydrazone) (Sigma) served as a positive destaining control. Cells were analyzed using a FACS Calibur flow cytometer, and data were analyzed using Cell-Quest Pro software (BD Bioscience). Reduction of TMRE mean fluorescence of viable cells (FSC/SSC) was analyzed. Untreated controls were set as 100%.

**Immunoblotting**

NK cells were lysed in B++ lysis buffer (20 mM HEPES, pH 7.5, 0.4 M NaCl; 1 mM EDTA, 1 mM EGTA, 1 mM DTT) supplemented with mini complete protease inhibitor cocktail tablet (Roche Diagnostics, Mannheim, Germany), sonicated, mixed with NuPAGE loading buffer (Invitrogen), boiled at 96°C*,* subsequently separated by sodium dodecyl sulphate-polyacrylamide gel electrophoresis (SDS-PAGE) and transferred to Hybond enhanced chemiluminescence (ECL) nitrocellulose membrane (Amersham Bioscience, Uppsala, Sweden). Membranes were incubated with anti-CD3ζ-horseradish peroxidase antibody (HRP; Santa Cruz Biotechnology) according to the manufacturer’s protocol and stained with Ponceau S (Sigma). Chemiluminescence was used for visualization using the ECL Western blotting detection reagents (PerkinElmer) according to the manufacturer's instructions.


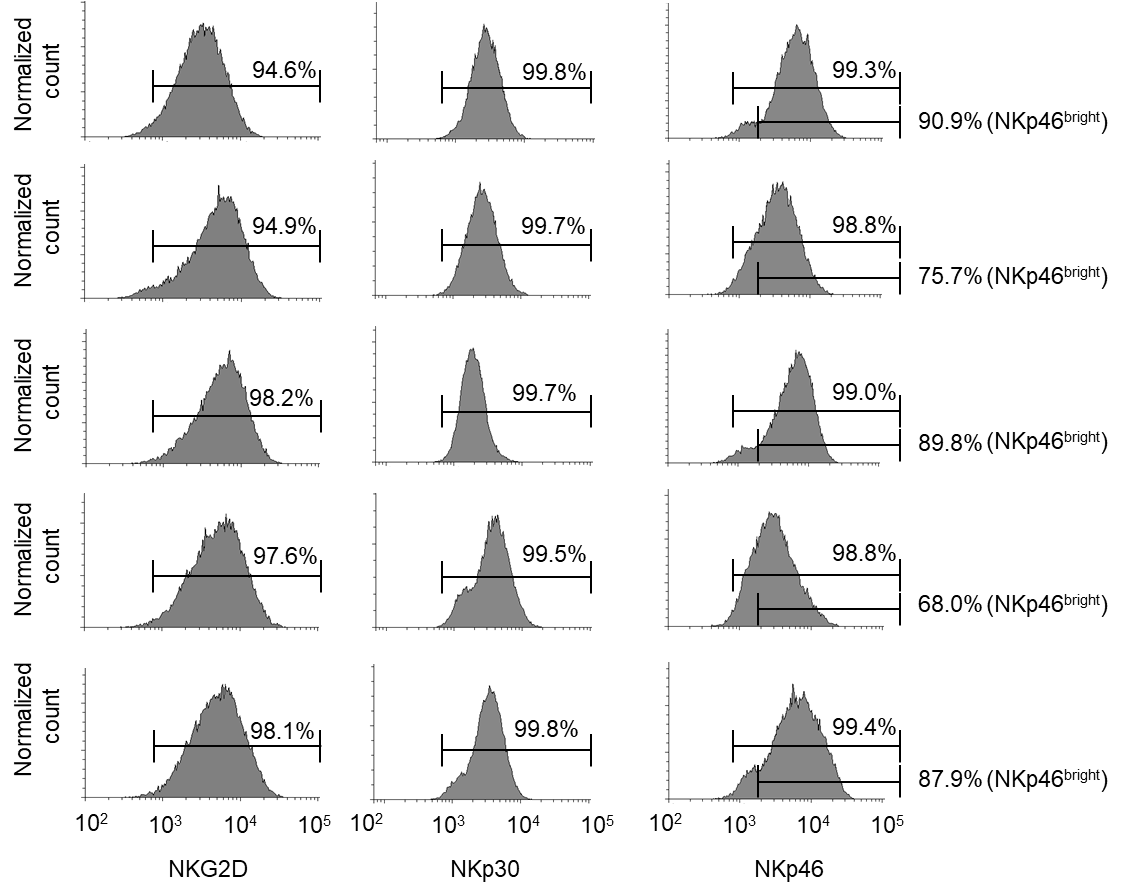


**Supplement Figure 1: Expression of NKp30, NKp46 and NKG2D in NK cells day 6-8 after isolation from 5 healthy donors.**


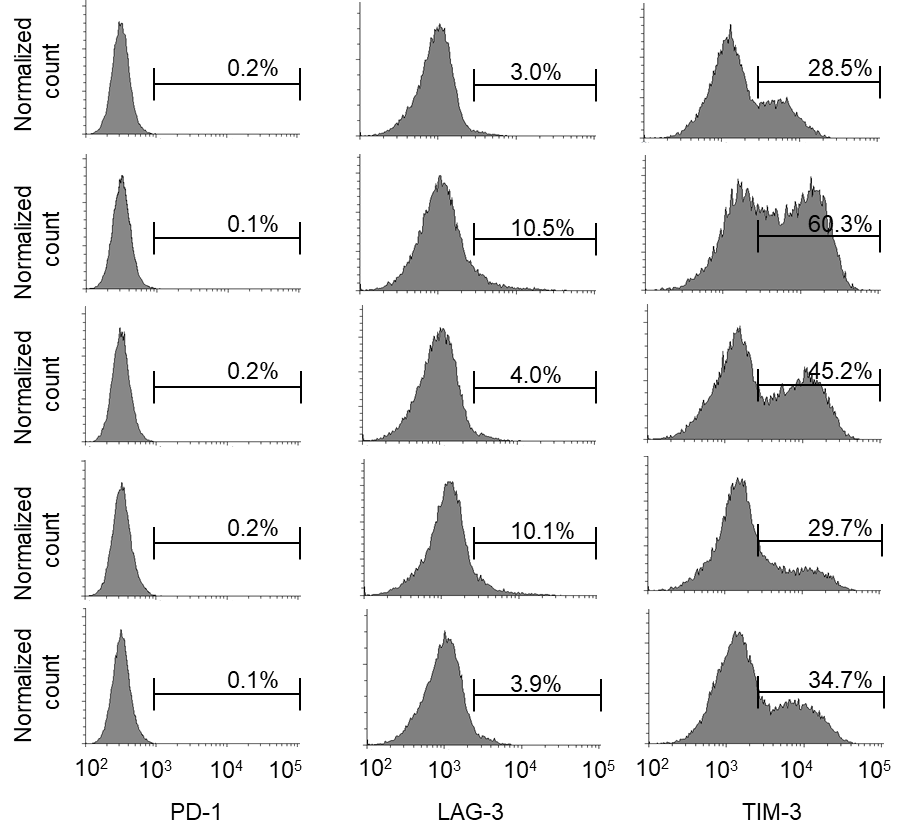


**Supplement Figure 2: Expression of PD-1, LAG-3 and TIM-3 in NK cells day 6-8 after isolation from 5 healthy donors.**

**
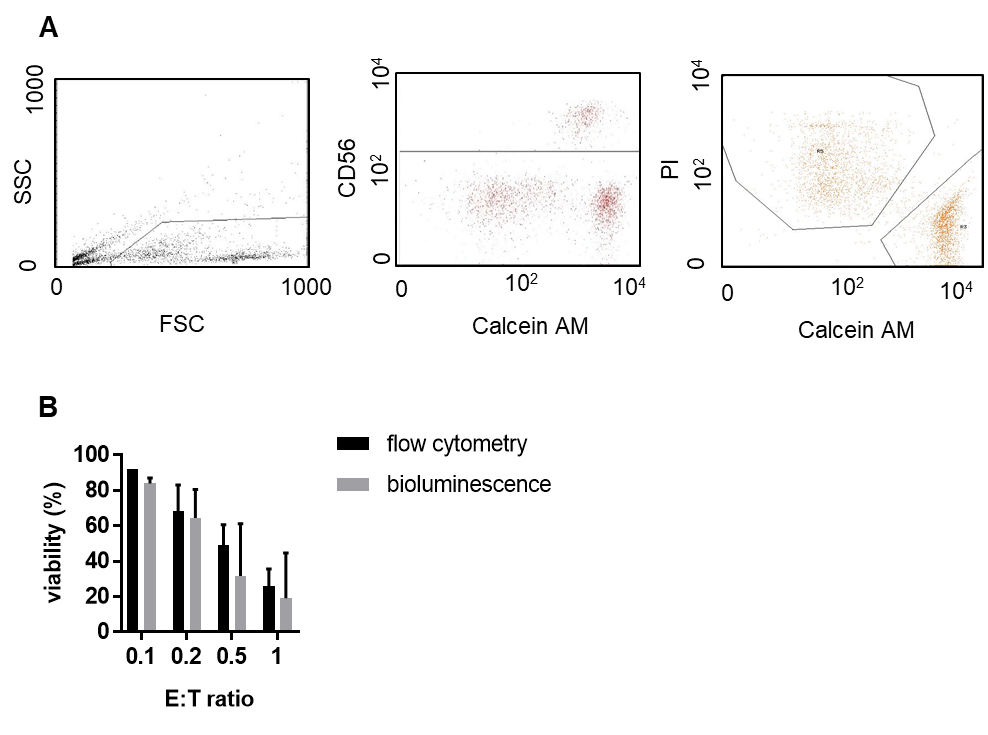
**

**Supplement Figure 3: Viability assessment of ALL5 PDX cells after 24 hours of coculture with aCD19 CAR NK cells at different E:T ratios. A)** Gating strategy for flow cytometric analysis of CD56-/Calcein AM+ /PI- cells. **B)** Flow cytometric (% CD56-/Calcein AM+/PI- cells) or bioluminescent readouts for cell viability.


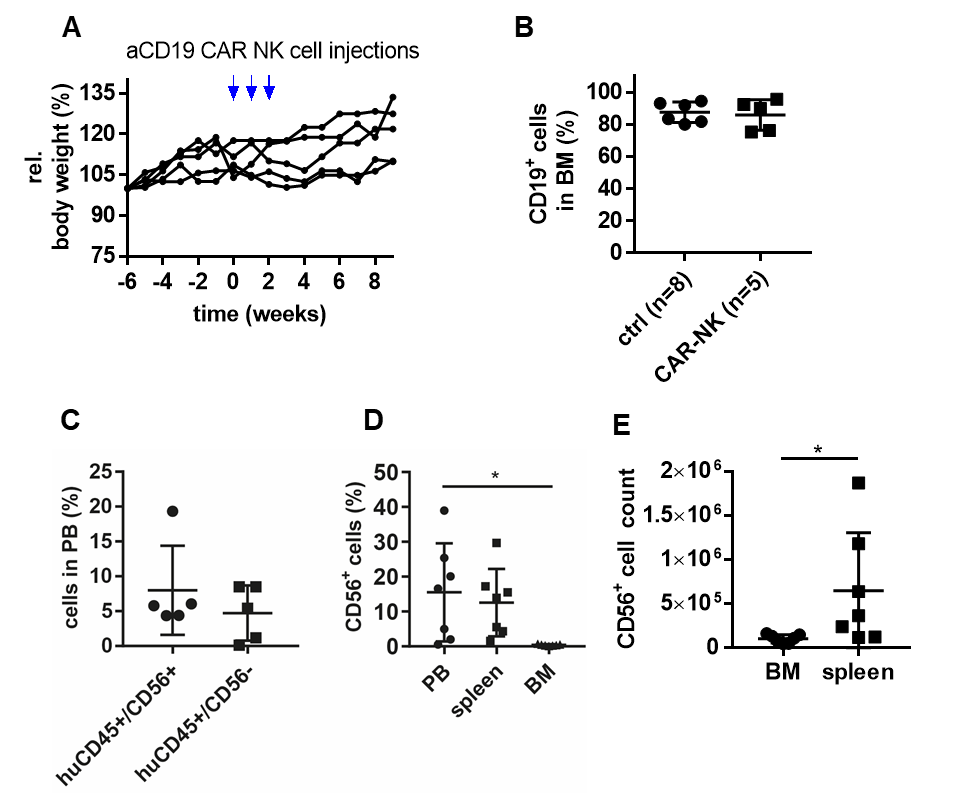


**Supplement Figure 4: Analysis of body weight and NK cells in PB, spleen and BM of NSG mice.** A) Body weight of ALL6-transplanted NSG mice treated with 3 injections of aCD19 CAR NK cells (indicated by blue arrows). B) Proportion of CD19+ cells (PDX cells) in bone marrow of sacrificed mice. C) Amount of leukemic (CD45^+^/CD56^-^) and CAR NK cells (CD45^+^/CD56^+^) of total viable nucleated cells 4-5 days post second CAR NK cell application in peripheral blood of ALL6-transplanted NSG mice. D,E) Distribution of NK wells in NSG mice. Mice were sacrificed 4-5 days after second NK cell application (n=7). Cells were isolated from PB, spleen and bone marrow (both hind limbs) and subsequently analyzed via flow cytometry for (D) percentage of CD56+ cells or (E) total cell count of all isolated viable nucleated cells. * p<0.05, one-way ANOVA with Bonferroni post hoc test.


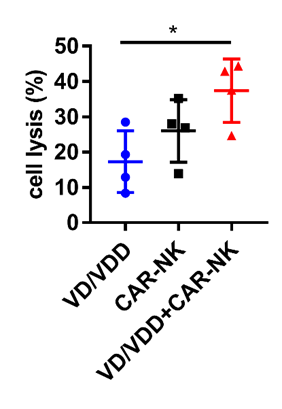


**Supplement Figure 5: Simultaneous treatment of PDX cells with pharmacotherapy and aCD19 CAR NK cells *in vitro*.** BCR-ABL-negative PDX cells ALL2 and ALL4 were incubated with VEN/DEX (VD) and BCR-ABL-positive ALL6 and ALL8 with VEN/DEX/DAS (VDD) for 24 hours alone or in combination with aCD19 CAR NK cells in a E:T ratio of 0.1:1. Cytotoxicity was assessed using flow cytometric analysis of CD56-/Calcein AM+/PI- staining. Statistical significance was determined by one-way ANOVA with Bonferroni post hoc test. *p<0.05.


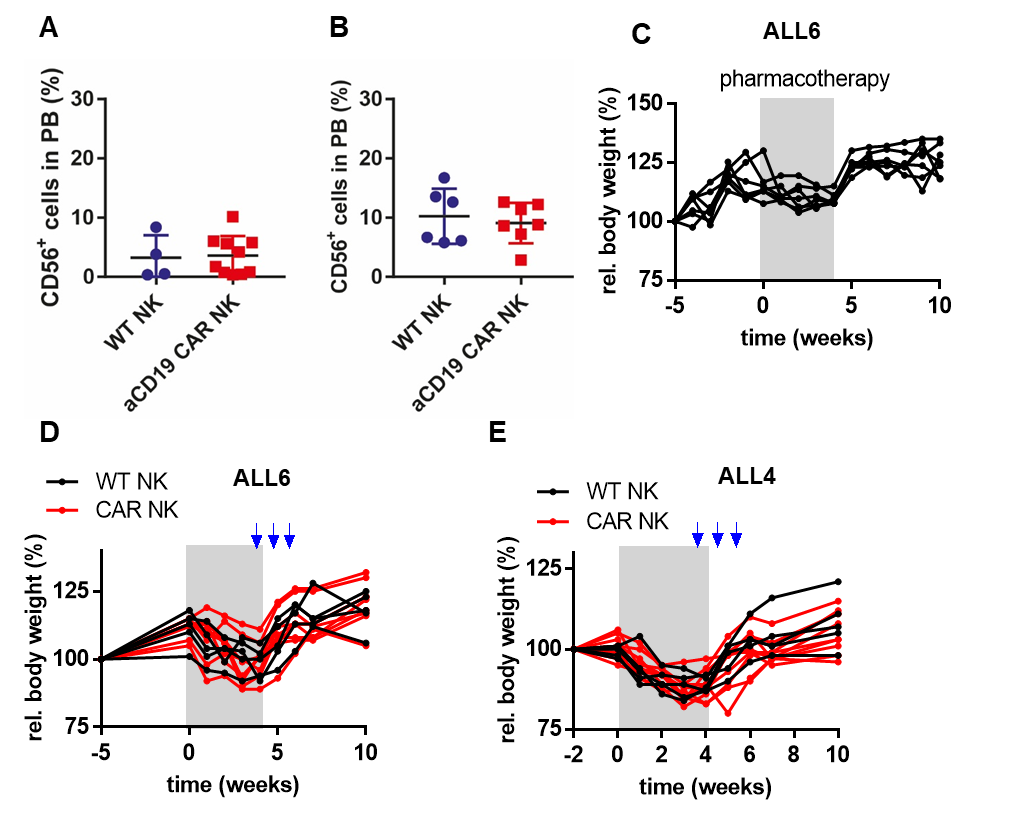


**Supplement Figure 6: CD56+ NK cells in PB and body weight of ALL PDX transplanted NSG mice.** A) ALL4- and B) ALL6-transplanted NSG mice were treated with induction pharmacotherapy followed by cellular therapy with either WT NK or aCD19 CAR NK cells. 5 days after the second NK cell application the amount of CD56+ NK cells of viable nucleated cells in peripheral blood was analyzed via flow cytometry. C-E) Body weight of ALL6 (C,D) and ALL4 (E) transplanted mice treated with pharmacotherapy alone (C) or with subsequent WT NK cell or CAR NK cell injections (D,E), respectively. Grey bars indicate the oral treatment period and the blue arrows indicate NK cell injections.


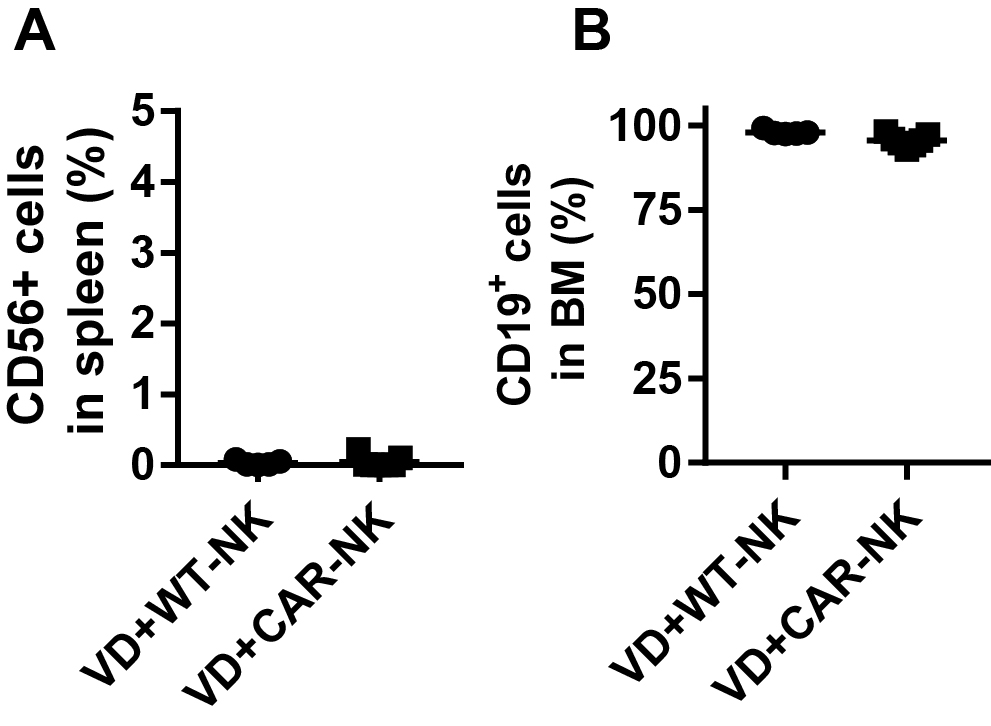


**Supplement Figure 7:** **Analysis of relapses from ALL4 NSG mice treated with pharmacotherapy and cellular immunotherapy.** (A-B) Flow cytometric analysis of CD56 and CD19 expression of isolated viable nucleated cells from (A) spleen and (B) bone marrow of relapsed mice (n=5).

**Supplement Table 1:** Characteristics of ALL patients used for PDX generation. PB or BM samples were used to generate PDX models in NSG mice. PDX ALL1-5 were previously published in (18), ALL10 and ALL11 are published in (42, 43), respectively.

| sample | diagnosis | initial diagnosis/ relapse | cytogenetics/molecular genetics | age | gender | immunophenotype |
| --- | --- | --- | --- | --- | --- | --- |
| ALL1 | pro-B-ALL | relapse | 46,XY,t(4;11)(q21;q23), KMT2A::AFF1 | 39 | male | 30% CD45dim blasts: CD19+, CD22+, partial cyCD79a+, CD34-, CD10-, CD20- |
| ALL2 | c-ALL | relapse | 55,XY,+1,dup(4)(q22.1q22.1),+6,+8,inv(8)(q21.1q24),del(9)(p21.3),+10,+11q,+14,+18,+19,+21,+X | 41 | male | 12% CD45dim blasts: CD19+, CD20+, CD22+, CD79a+, CD38, cyTdT+, partial CD10+, CD34- |
| ALL3 | c-ALL | relapse | 46,XY,der(1)t(1;19)(q23.3;p13.3),dup(1)(q23.3qter),der(6)t(6;7)(q12;21.3)del(6)(q12qter),dup(7)(q21.3qter)del(9)(p13.2) ,TCF3::PBX1 | 37 | male | 90% CD45dim blasts: CD34-, CD19+, CD10+, CD22+, CD24+, HLA-DR+, IgM-, cyTdT-, cyCD22-, cyCD79a-, cyMPO-, CD117-, cyCD3- |
| ALL4 | pro-B-ALL | relapse post HSCT | 46XY, t(11;19)(q23;p13) KMT2A::MLLT1 | 37 | male | 79% CD45dim blasts: CD19+, CD10-, HLA-DR+, CD15+, cyCD79a+, CD22+ |
| ALL5 | c-ALL | initial diagnosis | 45XY,-9,t(9;22)(q34;q11) BCR-ABL p190 | 70 | male | 49% CD45dim blasts: CD19+, CD10+, CD24+, HLA-DR+, CD34+, partial CD20+, partial CD22+, partial CD13+, partial CD15+ |
| ALL6 | lymphatic blastcrisis CML | relapse post HSCT | complex karyotype 46-48 XY, t(9;22), +8, del(8)(p11), der(8;12)x1-2,i(8)(q10), +12, +17, ABL-kinase mutation c.703A>G; p.Met244Val (Imatinib resistance) B2a2) | 39 | male | 54% CD45dim blasts: CD34+, CD19+, CD10+, CD24+, CD123+, HLA-DR+, cyTdT+, cyCD79a+ |
| ALL7 | c-ALL | initial diagnosis | 51,XX,+X,+X,+4,del(7)(p12p15),add(9)(p12),t(9;22)(q34;q11),+14,+der(22)t(9;22) | 68 | female | 28% CD45-/CD34+ blasts and 44% CD45dim/CD34+ blasts, : CD19+, CD10+, CD22+, CD24+, HLA-DR+, cyTdT+, cyCD79a+, CD20+ |
| ALL8 | c-ALL | initial diagnosis | t(9;22) BCR-ABL | 59 | male | 28% CD45-/CD34+ blasts and 52% CD45dim/CD34+ blasts: CD19+, CD10+, CD24+, cyTdT+, cyCD79a+, partial CD20+, CD13+, CD33+, CD14+, HLA-DR+, CD123+ |
| ALL9 | c-ALL | relapse | 46,XY,t(14;16)(q23;q24)[12]/53,idem,+X,+Y,+der(1;5)t(1;5)(p11;q35),+6,-9,+10,der(19)t(19;20)(q13;q11),del(20)(q11),+21,+21,+22[9] | 20 | male | CD34+, CD19+, CD10+, partial CD20+, CD22+, partial CD24+, HLA-DR+, C79a+, CD38+, CD123+, CD13+ |
| ALL10 | c-ALL | initial diagnosis | t(17;19)(q21-q22;p13) TCF3::HLF | 16 | female | CD19+, CD10+, CD20–, CD34– |
| ALL11 | c-ALL | initial diagnosis | t(9;22) BCR-ABL | 13 |  | CD19+, CD10+, CD20+, CD34+ |

**Supplement Table 2**: PDX cell characteristics. Immunophenotype, cell viability after thawing, background of murine CD45+ cells in samples used for further studies and amount of human CD45+ cells expressing CD19 are shown.

| sample | immunophenotype PDX | viability (%) | murine CD45+ cells (%) | CD19+ of human cells (%) |
| --- | --- | --- | --- | --- |
| ALL1 | CD19+, CD10-, CD20-, CD22+ | 60 | 2.51 | 99.6 |
| ALL2 | CD19+, CD10-, CD20+, CD79a+, CD38+,CD34+ | 58 | 5.48 | 98.6 |
| ALL3 | CD19+, CD10+, CD22+, CD24+, HLA-DR+ | 82 | 1.95 | 99.3 |
| ALL4 | CD19+, CD10-, CD22+, HLA-DR+ | 86 | 0.91 | 99.9 |
| ALL5 | CD19+, CD10+, CD24+, HLA-DR+, CD34+, CD22+, partial CD13+ | 90 | 3.58 | 100 |
| ALL6 | CD19+, CD10+, partial CD24+, CD123+, HLA-DR+ | 87 | 3.09 | 99.9 |
| ALL7 | CD19+, CD10+, CD22+, CD24+, HLA-DR+, CD20+, CD34+ | 69 | 10.13 | 97.7 |
| ALL8 | CD19+, CD10+, CD24+, CD20+, CD13+, CD33+, HLA-DR+, CD123+, CD22+ | 85 | 4.89 | 99.8 |
| ALL9 | CD19+, CD10+, CD20-, CD22+, CD34+, CD24+, HLA-DR+, CD79a+, CD38+, CD123+, CD13+ | 82 | 2.1 | 99.7 |
| ALL10 | CD19+, CD10+, CD20+, CD34- | 74 | 4.51 | 99.9 |
| ALL11 | CD19+, CD10+, CD20+, CD34+ | 93 | 4.84 | 99.5 |

**Supplement Table 3:** NK cell subset analysis of the respective donors upon NK cell isolation.

| **donor** | **CD56^bright^CD16^-^** **cells (%)** | **CD56^+^CD16^+^** **cells (%)** | **CD56^dim^CD16^-^** **cells (%)** |
| --- | --- | --- | --- |
| 1 | 1.78 | 96.75 | 1.43 |
| 2 | 1.85 | 97.87 | 0.25 |
| 3 | 4.83 | 94.75 | 0.45 |
| 4 | 2.52 | 95.43 | 2.00 |
| 5 | 2.31 | 94.75 | 0.88 |
| 6 | 2.28 | 96.98 | 0.73 |
| 7 | 3.29 | 95.97 | 0.75 |
| 8 | 3.43 | 95.69 | 0.84 |
| mean | 2.96 | 95.84 | 0.94 |
| SD | 1.08 | 1.18 | 0.53 |

**Supplement Table 4:** NK cell subsets from three donors upon NK cell isolation and after 6 days in culture with hIL-2 and hIL-15.

| **population** | **CD56^bright^CD16^-^**  **cells (%)** | **CD56^+^CD16^+^**  **cells (%)** | **CD56^dim^CD16^-^**  **cells (%)** |
| --- | --- | --- | --- |
| Isolation | 2.76 (±1.64) | 95.97 (±2.39) | 1.25 (±0.92) |
| Cultivation | 24.78 (±21.34) | 71.34(±18.41) | 4.25 (±1.48) |

**Supplement Table 5:** Drug concentrations of VEN, DEX and DAS used for *in vitro* pretreatment of PDX cells.

| sample | VEN (nM) | DEX (nM) | DAS (nM) |
| --- | --- | --- | --- |
| ALL4 | 20 | 20 | - |
| ALL2 | 100 | 100 | - |
| ALL11 | 50 | 50 | 0.25 |
| ALL5 | 100 | 100 | 0.5 |
| ALL8 | 200 | 200 | 20 |
| ALL6 | 50 | 100 | 0.5 |

**Supplement Table 6:** Time of relapse of ALL6 transplanted mice treated with VDD+WT NK or VDD+CAR NK.

|  |  | sacrificed | | |
| --- | --- | --- | --- | --- |
| mouse | treatment group | days after transplantation | days after stop of VDD treatment | days after last NK cell injection |
| 1 | untreated leukemia control | 119 | - | - |
| 1 | VDD+WT NK | 294 | 237 | 218 |
| 2 | VDD+WT NK | 327 | 270 | 230 |
| 3 | VDD+WT NK | 322 | 248 | 239 |
| 1 | VDD+CAR NK | 368 | 311 | 278 |
| 2 | VDD+CAR NK | 280 | 220 | 204 |
| 3 | VDD+CAR NK | 329 | 269 | 253 |

**Supplementary References**

39. Sarikidi A, Kefalakes E, Falk CS, et al. Altered Immunomodulatory Responses in the CX3CL1/CX3CR1 Axis Mediated by hMSCs in an Early In Vitro SOD1(G93A) Model of ALS. Biomedicines. 2022;10(11).

40. Sandrin V, Boson B, Salmon P, et al. Lentiviral vectors pseudotyped with a modified RD114 envelope glycoprotein show increased stability in sera and augmented transduction of primary lymphocytes and CD34+ cells derived from human and nonhuman primates. Blood. 2002;100(3):823-32.

41. Suerth JD, Morgan MA, Kloess S, et al. Efficient generation of gene-modified human natural killer cells via alpharetroviral vectors. J Mol Med (Berl). 2016;94(1):83-93.

42. Pal D, Blair HJ, Elder A, et al. Long-term in vitro maintenance of clonal abundance and leukaemia-initiating potential in acute lymphoblastic leukaemia. Leukemia. 2016;30(8):1691-700.

43. Rehe K, Wilson K, Bomken S, et al. Acute B lymphoblastic leukaemia-propagating cells are present at high frequency in diverse lymphoblast populations. EMBO Mol Med. 2013;5(1):38-51.
